# Reframing TB Care: A Perspective on Multimorbidity-Centered Care for People with TB

**DOI:** 10.3390/tropicalmed11020037

**Published:** 2026-01-29

**Authors:** Alexa Tabackman, Sadie Cowan, Claire Calderwood, Pranay Sinha

**Affiliations:** 1 Section of Infectious Diseases, University of Pennsylvania, Philadelphia, PA 19104, USA; 2 Division of Infectious Diseases, Massachusetts General Hospital, Boston, MA 02114, USA; 3 National Heart and Lung Institute, Imperial College London, London SW3 6LY, UK; claire.calderwood2@lshtm.ac.uk; 4 Clinical Research Department, London School of Hygiene & Tropical Medicine, London WC1E 7HT, UK; 5 The Health Research Unit Zimbabwe, Biomedical Research and Training Institute, Harare P.O. Box A178, Zimbabwe; 6 Boston Medical Center, Boston, MA 02118, USA; psinha@bu.edu; 7 Section of Infectious Diseases, Boston University Chobanian & Avedisian School of Medicine, Boston University, Boston, MA 02118, USA; 8 Joan Klein Jacobs Center for Precision Nutrition and Health, Cornell University, Ithaca, NY 14850, USA; 9 Center on Emerging Infectious Diseases, Boston University, Boston, MA 02118, USA

**Keywords:** tuberculosis, multimorbidity, multidisciplinary care

## Abstract

Tuberculosis (TB) rarely occurs in isolation; most people with TB experience multiple coexisting conditions, including HIV, diabetes, undernutrition, depression, and substance use disorders, which worsen disease severity and compromise treatment outcomes. Although the World Health Organization has issued disease-specific guidance for managing key comorbidities, TB care remains largely siloed and poorly equipped to address the growing burden of multimorbidity, particularly in African health systems. In this perspective article, we propose a phased framework for multimorbidity-centered TB care. The first phase emphasizes systematic screening for common comorbidities and establishment of basic referral pathways. The second phase focuses on strengthening coordination between TB programs and existing health and social services, including task sharing and longitudinal follow-up. The third phase advances toward fully integrated, co-located, multidisciplinary models of care that embed TB services within broader multimorbidity platforms. Together, this framework offers a pragmatic roadmap for TB programs to deliver more person-centered, equitable, and efficient care, strengthen primary care systems, and accelerate progress toward ending TB as a public health threat in Africa.

## 1. Introduction

In 2024, an estimated 10.7 million people fell ill with tuberculosis (TB) and 1.23 million died despite the widespread availability of preventative strategies and effective treatments [1]. The leading drivers of TB incidence and mortality worldwide are undernutrition, HIV, smoking, alcohol use disorders and diabetes [1]. Each of these vulnerabilities and others, including mental health conditions such as depression, are also associated with more severe disease and worse treatment outcomes [1,2,3,4,5]. TB itself can additionally exacerbate these conditions, perpetuating a vicious cycle and contributing to increased morbidity and mortality from TB as well as morbidity and mortality many years after treatment completion [6,7,8,9]. These conditions shape TB risk and outcomes through both biological mechanisms and socioeconomic pathways [3,10,11,12].

Multimorbidity, typically defined as the coexistence of two or more chronic health conditions in an individual, is increasing in prevalence in low- and middle-income countries [13,14]. This trend is particularly relevant for TB care, as growing numbers of people with TB also live with additional chronic conditions [15,16,17,18]. A secondary analysis of WHO Global Health Survey data suggests that up to two-thirds of people with TB have at least one non-communicable disease, while approximately two-fifths have two or more [19]. Consistent with this, a study from Brazil found that people with TB and two or more comorbid conditions experience roughly double the mortality and lower treatment success compared with those without multimorbidity [20]. Together, these patterns suggest that rising multimorbidity is likely to drive higher healthcare utilization, greater costs, and worse clinical outcomes, even as substantial gaps remain in accurately estimating the prevalence of multimorbidity among people with TB [8,9,16,18,19,20].

The current WHO framework for TB and comorbidities provides essential guidance for managing key conditions including HIV, diabetes, undernutrition, mental health disorders, tobacco use, and alcohol use among people with TB [21]. While these guidelines emphasize bidirectional screening, collaborative care, and integration of comorbidity management within TB services, they remain largely disease-specific and implementation lags, particularly in African countries [22]. Indeed, evidence supporting multimorbidity care for people with TB has outpaced its implementation at scale [10,23,24].

Here, we argue that reorienting TB services around multimorbidity care acknowledges the shared biological and social determinants linking TB and chronic conditions, their reinforcing effects, and the lived experience of people managing multiple, intersecting health challenges. We define multimorbidity care for people with TB as a holistic model that provides integrated service delivery for PWTB and comorbid conditions. The proposed benefits of this approach are providing person-centered care that addresses the health conditions that can negatively impact care outcomes for PWTB and streamlining delivery of healthcare services. Applying this framing to TB care requires operational changes: moving from parallel disease-specific services to integrated systems that can identify and manage co-occurring conditions in routine practice. In the sections that follow, we describe the current context of multimorbidity care for PWTB and outline a phased framework for developing such an integrated model to better define and address multimorbidity among PWTB in Africa.

## 2. Current Context of Multimorbidity Care for PWTB

The experience of HIV-TB integration in Africa provides an important example of how additional services might become a routine component of TB care. This integration of services has been associated with improved uptake of antiretroviral therapy, retention in care, and TB outcomes, and is estimated to have saved 9 million lives in the period 2005–2020 [6,25,26,27,28,29]. There is also evidence of efficiency cost-savings [30]. Nonetheless, TB/HIV integration took a long time to become established and remains incomplete despite strong, longstanding endorsement in policy and guideline documents, political buy-in, dedicated funding and inclusion in routine monitoring and reporting frameworks. Even in countries such as South Africa that have adopted a model of co-located HIV and TB care at the primary care level there remain significant challenges in implementing integrated service delivery [31,32,33,34].

Although conditions such as undernutrition, substance use disorders, mental health conditions, and diabetes are highly prevalent among people with TB and have been acknowledged in policy for over two decades, they have received less emphasis in integrated service delivery. While this may partly reflect weaker biological associations with TB mortality compared with HIV (e.g., 19-fold higher mortality among PLHIV as compared to four-fold among people with severe undernutrition and three-fold higher among people with diabetes), this landscape is changing as long-term ART improves survival for millions, non-communicable diseases expand without commensurate expansion in treatment options, and climate change exacerbates global nutritional vulnerability [10,11,19,35,36,37].

Across many African countries, implementation of integrated TB services is constrained by barriers at multiple levels of the health system. In a 2022 survey of TB providers from 27 high-burden countries, HIV was consistently addressed in TB guidelines and linked to routine screening and referral, whereas other common comorbidities, including diabetes, tobacco use, alcohol use, and depression, were far less frequently incorporated into policy or practice [22]. These findings highlight persistent gaps between WHO recommendations and national TB guidelines, underscoring the need for policy shifts to support systematic screening and referral for non-HIV comorbidities [22,38]. The lack of consistent comorbidity screening and care recommendations in policy guidelines additionally perpetuates siloed funding streams that prioritize disease-specific programs and maintain vertically integrated systems of care rather than horizontally integrated programs that acknowledge the shared social and biologic determinants that underlie TB multimorbidity [31,32,39].

Screening and referral to services has been the most commonly implemented approach to integrating care. However, service delivery remains fragmented, with TB and comorbidity care often provided at separate facilities. This fragmentation limits data sharing across systems and places substantial burdens on individuals, who must navigate multiple points of care even when referral mechanisms exist. As a result, the risk of loss to follow-up remains high [40,41,42,43,44]. Co-located services at a single facility offer a more person-centered model of care but require substantial investment in infrastructure and provider training. Another alternative is to provide community-based management for co-morbidities with the assistance of community health workers, but effective implementation requires dedicated investment in training and supervision [45].

Integration is particularly challenging for comorbidities such as substance use disorders and mental health conditions, where limited provider capacity and weak referral pathways constrain the capacity to intervene on these comorbidities even when they are identified through screening. In such settings, TB programs may rationally deprioritize screening for conditions they cannot reliably treat or refer. Qualitative analyses of TB/HIV programs in South Africa illustrate how this dynamic plays out in practice. Program managers identified structural constraints, including under-resourced mental health services, limited workforce training, and insufficient programmatic support as factors that undermined referral and follow-up even when mental health needs were recognized among PWTB [46]. Similarly, despite the identification of undernutrition, there are gaps in welfare programs that can address food insecurity among TB-affected households [47].

Numerous structural barriers, many of which are outlined above, have limited the effective implementation of multimorbidity management for people with TB. These challenges are compounded by substantial heterogeneity in TB incidence, the prevalence and mix of comorbidities, and the capacity of health systems across and within countries, making a single, standardized model of care impractical [35,48]. In response, we propose a pragmatic, phased approach to multimorbidity-centered TB care that allows programs to build capacity incrementally and adapt to local contexts. This approach aims to improve TB morbidity and mortality while ensuring more person-centered, integrated management for people living with TB while also ensuring a more efficient utilization of healthcare resources [49].

## 3. A Phased Implementation of Multimorbidity Care for PWTB

**Figure 1:**
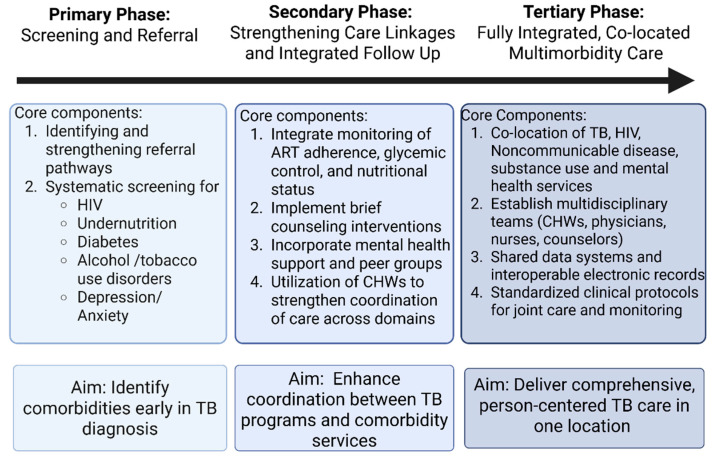
Phases of development of multimorbidity model for PWTB.

In this section, we build on the logic set out in prior work by Foo et al., extending their systematic review of TB–NCD integration efforts to develop a phased framework for integrated service delivery tailored to multimorbidity and the African context [24] (Figure 1).

### 3.1. Phase 1: Screening and Referral to Care

Early identification of comorbidities in persons with newly diagnosed TB and identifying referral pathways is a crucial first step in defining multimorbidity burden and developing integrated care. This should be the focus of the first phase of multimorbidity interventions. Much of this approach is grounded in existing WHO guidance from the TB and comorbidities framework and the 2024 Module 6 updates on HIV–TB and diabetes–TB care [50].

Timely recognition of HIV and implementation of ART is necessary to prevent mortality. Moreover, given the risk of paradoxical IRIS, HIV screening is critical for safe and effective TB treatment. Low-cost rapid diagnostic tests for HIV are now widely available in most African countries and ART services have been widely devolved to the primary health care level [51]. However, opportunities for improvement remain. All PWTB diagnosed with HIV should be promptly referred for initiation of ART and programs should track the proportion initiated and retained through treatment completion. Tracking ART uptake and adherence can highlight gaps in TB–HIV integration and guide quality improvement. Untapped resources such as community health workers can help further improve implementation of standardized early screening and management of co-prevalent HIV infections [45].

Nutritional assessment is a core component of TB care and is recommended by the WHO at diagnosis and throughout treatment [21]. Despite substantial heterogeneity in how nutritional status is measured across settings, simple, validated tools such as body-mass index and mid–upper arm circumference can be readily and inexpensively implemented by clinic staff [52,53]. Those with severe undernutrition may benefit from initial hospitalization for nutritional rehabilitation and close monitoring for treatment toxicity [54]. Beyond baseline assessment, longitudinal weight monitoring is essential, as early weight gain is a key marker of treatment response, while failure to gain weight is associated with poor prognosis and should prompt evaluation for poor adherence, drug resistance, or inadequate drug exposure [55,56]. Individuals who remain undernourished at the end of treatment should be followed up closely given their higher risk of recurrent disease and connected to available welfare programs. Together, these considerations underscore the need for consistent nutritional monitoring and clear triage pathways throughout TB treatment.

Routine screening for diabetes using point-of-care hemoglobin A1c or blood glucose testing should be performed for all PWTB. Integrated management of TB and diabetes remains challenging in many settings because of siloed services and limited provider familiarity with co-management, therefore clear referral pathways should be created for management of PWTB with diabetes [57]. Early recognition and management are critical, as diabetes is associated with higher risks of mortality, treatment failure, and relapse among people with TB, particularly in the setting of poor glycemic control [58,59]. In addition, TB can impair glucose tolerance, and rifamycins may reduce the effectiveness of some oral antihyperglycemic agents, underscoring the importance of early diabetes recognition to guide treatment adjustments [12,60].

Identifying comorbid nicotine dependence and alcohol use disorder among people with TB is important, as both are associated with poor treatment adherence and increased risk of loss to follow-up [61,62]. Screening for tobacco use can be readily implemented through routine assessment of smoking history and smoke exposure. Alcohol use disorder can be screened at treatment initiation using validated tools such as the Alcohol Use Disorders Identification Test–Concise (AUDIT-C) or the CAGE (Cut down, Annoyed, Guilty, Eye-opener) questionnaire [63,64]. Incorporating substance use screening with brief intervention and referral to treatment (SBIRT) into routine TB care represents a low-cost and feasible approach to addressing alcohol and tobacco use [65,66]. While time constraints may limit the provision of counseling within TB clinics, positive screening should prompt referral to available community and social services for counseling and treatment.

PWTB can be readily screened for depression and anxiety with validated tools such as the Patient Health Questionnaire-9 (PHQ-9) and Generalized Anxiety Disorder-7 (GAD-7) or other country-specific questionnaires [67,68]. These assessments can be administered by both clinicians and lay health workers. Because TB care involves sustained contact over at least six months, programs have a natural opportunity for longitudinal mental health engagement. We therefore recommend screening all PWTB at treatment initiation with repeat symptom monitoring at follow-up appointments.

Limited availability of referral services remains a major barrier to linkage to care for people with TB. The burden of comorbidities and the capacity of referral pathways vary widely across TB-endemic settings, with particularly pronounced gaps for alcohol use disorder, mental health conditions, and undernutrition. In these contexts, systematic screening retains value not only for individual care but also for identifying service gaps, informing resource allocation, and supporting advocacy to strengthen referral pathways.

### 3.2. Phase 2: Strengthening Care Linkages and Integrated Follow Up

The second phase should focus on strengthening linkages between tuberculosis programs and existing health and social services, making these services more TB-sensitive, and developing new service pathways where gaps exist. This phase emphasizes integrating key management steps within TB clinics and buttressing referral pathways through incremental capacity-building that leverages the sustained contact that TB programs have with PWTB.

Monitoring ART adherence, glycemic control, and weight throughout the treatment process provides important metrics for HIV and diabetes management and can be incorporated as a component of routine assessment during follow-up visits. TB clinicians should track medication adherence and be vigilant for evidence of treatment failure due to subtherapeutic drug exposure which occurs at higher rates among PWTB with diabetes, undernutrition, and those living with HIV [12,69,70]. Given that PWTB regain lost weight, often rapidly, during the intensive phase, tracking their weight during follow-up visits is also important to ensure adequate dosing of anti-tubercular drugs. Similarly, it is key to ensure that drug–drug interactions between anti-tubercular drugs and those used for HIV and diabetes are not overlooked. Strengthening referral pathways and communication between TB clinicians and other providers through means such as shared patient registries and standardized referral forms is a key component to aid in co-management of these conditions.

Ongoing screening for tobacco and alcohol use disorders throughout treatment, in addition to baseline screening, can be readily implemented. Positive screening for active tobacco use should prompt providers to offer counseling to aid in tobacco cessation. Brief counseling interventions such as the “5 As” (Ask, Advise, Assess, Assist, Arrange) and ABC (Ask, Brief, Cessation support) methods can also be instituted with minimal training to help PWTB decrease tobacco use [66,71]. A cluster RCT in North India found that incorporating the ABC package increased smoking cessation rates compared to standard of care (adjusted incidence risk ratio 1.56, 95% CI 1.24–1.93, *p* < 0.0001) and also demonstrated feasibility of incorporating tobacco cessation into standard TB treatment [72].

Mental health management should be a core component of TB management. The WHO operational handbook on tuberculosis and the WHO Mental Health Gap Action Programme framework provide practical guidance for screening, stepped care, and brief psychological interventions by trained non-specialists when referral options are limited [73,74]. In addition to counseling interventions, social support systems also play a strong role in addressing distress and stigma among PWTB. Peer support groups, such as the “TB clubs” in Ethiopia, where members meet regularly to share lived experiences and support treatment adherence, as well as other peer support systems, have been effective in reducing stigma and depression among people with TB with minimal demands on health system resources [75,76,77,78].

Nutritional counseling is now recommended by the updated WHO TB-Nutrition guidelines [50]. Impoverished PWTB often rely on carbohydrate-rich diets with low dietary diversity, putting them at risk of nutritional deficiency and poor glycemic control [79]. When performed effectively counseling can help improve consumption of locally available nutrient-rich foods in sufficient quantities and dispel dietary misconceptions [47,80]. Nutritional supplementation can improve adherence and likely leads to improved treatment outcomes and mortality [81,82]. While some TB-endemic regions have food assistance programs, PWTB have still have unmet caloric and micronutrient needs. Developing targeted food support, such as TB-specific food baskets or ready-to-use therapeutic foods, should be prioritized in collaboration between TB programs and social services [83].

Community health workers may be pivotal to implementing the secondary phase, serving as the bridge between PWTB, clinics, and community services [45]. They can monitor ART adherence and glycemic control during DOT encounters, deliver brief interventions for tobacco and alcohol use, provide nutritional and mental health counseling, and facilitate peer-support groups. By maintaining regular contact with PWTB and households, community health workers are uniquely positioned to identify early signs of treatment interruption, clinical deterioration, or psychosocial distress and to trigger timely referral or provider follow-up. To support this model, formal referral protocols, continuous capacity-building, and close coordination among TB programs, local health systems, and social service agencies are essential. Embedding community health workers within a multidisciplinary team allows a more person-centered, longitudinal approach to TB multimorbidity care while strengthening the overall health system.

Together, these efforts move TB programs beyond identification of comorbidities toward sustained, coordinated management. Unlike the initial phase, which emphasizes screening and referral, the secondary phase focuses on strengthening continuity of care through reliable linkages, shared responsibility across services, and ongoing follow-up over the course of treatment. This requires deliberate investment in workforce capacity, functional referral pathways, and routine communication between TB and comorbidity services to ensure that early integration translates into meaningful, durable improvements in care delivery.

### 3.3. Building Fully Integrated Multimorbidity Care

The tertiary phase should build upon the preceding integration efforts, moving towards the establishment of fully integrated, co-located models of care that address the full spectrum of health and social needs among PWTB. In this phase, TB care is embedded within a broader multimorbidity platform that addresses the full spectrum of clinical and social needs among people with TB, including prevention, treatment, and social support, within a single care setting.

Unlike the secondary phase, which relies on strengthened linkages and follow-up across services, the tertiary phase minimizes the need for external referral by co-locating essential services and establishing shared clinical protocols. This model envisions a multidisciplinary system encompassing CHWs, pharmacists, nurses, physicians, and counselors who can jointly assess, manage, and follow PWTB across comorbidities and minimizes the need for external referral by co-locating essential services and establishing clear clinical protocols for shared care. Operationally, this may involve expanding the scope of TB clinics or transitioning TB management into strengthened primary care platforms where individuals can access care for TB, its comorbidities, and other chronic conditions within a unified system.

Evidence supporting the feasibility of such integration is emerging. A prospective study in Pakistan demonstrated that integrating mental health counseling into TB services improved treatment completion among symptomatic individuals (92.9% vs. 75.1%), illustrating the potential of task-shared psychosocial care within TB clinics [84]. Similarly, the IMPACT study in Russia also demonstrated the effectiveness of physician-led integration of AUD treatment into TB care [64]. However, we are still far from developing an inclusive model in which PWTB can access care for multiple conditions, as well as readily available support services such as cash transfers and food supplementation within the clinic.

Although such fully integrated, co-located models remain uncommon in TB-endemic settings, they are not purely aspirational in Africa. For example, the Gugulethu Community Health Centre in Cape Town provides comprehensive, free services spanning HIV care, TB treatment, diabetes management, chronic disease clubs, mental and palliative care, short-term admissions, and social support services within a single facility [85]. While not designed specifically as a TB multimorbidity program, such platforms demonstrate the feasibility of delivering multidisciplinary, person-centered care under one roof in high-burden settings, and illustrate the type of health system infrastructure upon which tertiary-phase integration could build.

While co-located, multidisciplinary care is feasible, scaling such models will require standardized clinical protocols for multimorbidity assessment at diagnosis and during follow-up, interoperable data systems that support shared records and outcome tracking, and substantial investment in workforce training and infrastructure. Crucially, sustained implementation depends on addressing vertical financing structures, as durable integration cannot be achieved while TB, non-communicable diseases, mental health, and social services remain supported through separate funding streams. The tertiary phase therefore represents not only a clinical transformation but also a health system and financing reform necessary to deliver truly person-centered, multimorbidity-focused TB care.

## 4. Conclusions

Eliminating tuberculosis requires more than diagnostic expansion and pharmacologic treatment alone; it demands a care model that reflects the complex realities of multimorbidity. Despite TB’s broad impact on health systems, care has remained largely siloed, with limited horizontal integration across disease programs. Many of the conditions that cluster with TB are shaped by the same structural forces, including poverty, limited education, and poor health literacy, and addressing them is essential to improving outcomes for people with TB. A control strategy focused narrowly on *Mycobacterium tuberculosis* risks tunnel vision, failing to meet the broader health needs of the key and vulnerable populations in which TB is concentrated and limiting the likelihood of durable reductions in TB burden without addressing the biological and social conditions that sustain transmission and disease progression.

While this Perspective focuses on well-recognized comorbidities that commonly intersect with TB, these conditions are not exhaustive. The burden and pattern of comorbidities relevant to TB vary substantially across settings, and multimorbidity-centered TB care must remain adaptable to local epidemiology. For instance, chronic kidney disease is of increasing concern as a driver and co-morbidity of TB in Africa. Similarly, locally prevalent occupational and environmental exposures, such as silicosis or berylliosis, may substantially shape TB risk in specific populations [86]. Together, these examples reinforce the need for flexible, context-specific integration strategies that can accommodate evolving evidence and locally relevant disease patterns rather than relying on uniform comorbidity checklists.

Multimorbidity management must therefore become a core component of TB control, supported by scalable interventions that extend beyond biomedical treatment to include nutrition, chronic disease management, and mental health care. The phased framework proposed here offers a pragmatic pathway toward this goal, progressing from screening and referral to fully integrated, co-located models of care. Implementing this approach will require alignment of policies, financing, and monitoring systems across disease programs, but such integration is both feasible and necessary. Embedding multimorbidity care within TB services provides an opportunity to move beyond fragmented delivery toward more equitable, efficient, and person-centered care, accelerating progress toward ending TB as a public health threat.
